# Evaluating a Smartphone App to Monitor Blood Pressure in Normotensive Pregnancies, High-Risk Pregnancies, and Women With Preeclampsia: Prospective Longitudinal Feasibility Study

**DOI:** 10.2196/70370

**Published:** 2026-02-18

**Authors:** Maria E Andersson, Christine Rubertsson, Elia Psouni, Lena Erlandsson, Camilla Edvinsson, Stefan R Hansson

**Affiliations:** 1 Department of Obstetrics and Gynecology, Skane University Hospital, Institution of Clinical Sciences L, Lund University, BMC l:12, Lund, 22184, Sweden, +46 46 17 25 07; 2 Department of Health Sciences, Skane University Hospital, Medical Faculty, Lund, Lund University, Lund, Sweden; 3 Department of Psychology, Lund, Lund University, Lund, Sweden; 4 Department of Obstetrics and Gynecology, Institution of Clinical Sciences Lund, Lund University, Lund, Skåne, Sweden

**Keywords:** blood pressure, experiences, mobile telephone, preeclampsia, self blood pressure monitoring, pregnancy

## Abstract

**Background:**

Antenatal care has been crucial in reducing maternal mortality. Currently, screening programs of pregnant women include blood pressure (BP) measurements, urine protein tests, and the identification of risk factors. Home monitoring can enhance the early detection and management of pregnancy-related hypertension, while also empowering women to take an active role in their own health care.

**Objective:**

This study aimed to evaluate the reliability and accuracy of contactless BP monitoring using the Anura smartphone app and to compare it to conventional manual cuff measurements. This was done in normotensive and high-risk pregnancies, as well as in women diagnosed with preeclampsia. A secondary objective was to assess women’s experience using the Anura app.

**Methods:**

Pregnant women with normotensive or high-risk pregnancies were enrolled from pregnancy weeks 8‐14, and women with preeclampsia were enrolled at the time of diagnosis. The 3 study groups consisted of 132 women with normotensive pregnancies, 40 women with high-risk pregnancies, and 87 women with preeclampsia. They were instructed to use the Anura smartphone app and perform a 30-second facial scan, alongside manual BP measurements, throughout pregnancy. Differences between the 2 methods were analyzed with linear mixed models accounting for repeated measures, reporting beta coefficients with 95% CIs, stratified by patient group and trimester. Outliers were detected visually in the Bland-Altman plots. A digital survey was answered in the Anura app at gestational weeks 37‐39, about their experiences using the Anura app.

**Results:**

A total of 4932 BP measurements were recorded with Anura, of which 539 had corresponding manual measurements. In normotensive pregnancies, Anura consistently showed slightly higher diastolic values (approximately 5‐7 mm Hg) and lower systolic values, with significant differences in the second and third trimesters. In high-risk pregnancies, both the systolic and diastolic BP were generally lower with Anura, especially in the second and third trimesters, while women with preeclampsia showed the largest differences, with Anura clearly showing lower systolic and diastolic values. Bland-Altman analyses confirmed these patterns and showed increasing variability and wider limits of agreement in the high-risk pregnancies with preeclampsia. Of 172 women with normotensive and high-risk pregnancies, 56 (32.5%) evaluated their experiences that were predominantly positive, with high perceived safety, better control, and a feeling of increased responsibility for their own health. Some experienced the measurement as somewhat uncomfortable.

**Conclusions:**

The Anura app is well accepted by pregnant women and supported them to take an active role in their own health care. Agreement with manual BP measurements was acceptable in normotensive pregnancies but lower in high-risk and preeclamptic pregnancies. These findings indicate potential for Anura as a complementary self-monitoring tool. Further development is needed to improve the app’s accuracy in high-risk groups before broader implementation can be recommended.

## Introduction

Antenatal care has been the most important health care intervention when it comes to reducing maternal mortality in modern times [1]. The current national antenatal care program in Sweden includes screening of pregnant women, performing blood pressure (BP) measurements, urine dipstick tests (proteinuria), and obtaining medical history to identify risk factors [2]. Home BP measurements by pregnant women using a validated normal standard BP cuff have shown lower BP levels than measurements at the clinic and a reduced need for antenatal care visits [3,4]. However, many pregnant women find this method to be uncomfortable and cumbersome, which might increase the risk of infrequent use and, consequently, failure to discover significant BP changes associated with such pregnancy risks as preeclampsia. Noncontact and cuffless BP measurement techniques have been studied in different populations with varying results. Studies show both acceptable accuracy and significant margins of error, and reviews point to methodological limitations, particularly in studies of pregnant women [5-8].

Preeclampsia is one of the most common causes of maternal and fetal mortality worldwide and affects 3‐8% of all pregnant women, corresponding to a total of 8.5 million women annually [9-11]. It is defined as hypertension at 140/90 mmHg after 20 weeks of gestation in combination with maternal organ dysfunction and/or intrauterine growth restriction [2]. Risk factors for preeclampsia, such as diabetes, chronic hypertension, kidney diseases, BMI >30, or African origin, are used as part of risk assessment in the first trimester of pregnancy [12,13] to provide prophylactic treatment with acetylsalicylic acid and reduce the risk of developing preeclampsia. Abnormal calcium and potassium levels have also been shown to be risk factors for pregnancy-related BP [14]. More complex screening algorithms, including biomarkers and Doppler ultrasound, such as the fetal medicine foundation model, have still not been evaluated in Sweden but recently in Denmark [15]. The fetal medicine foundation model, which combines maternal history factors with measurements of mean arterial pressure, uterine artery pulsatility index, and serum placental growth factor levels [16], is well validated but complex, requiring specially trained personnel to perform the Doppler ultrasound. Thus, it is essential to develop a more user-friendly method, available for all pregnant women for early identification of hypertension. A more straightforward and accessible BP monitoring solution would allow the women to be more involved in self-care and their health, both during pregnancy and postpartum.

**Figure 1.**
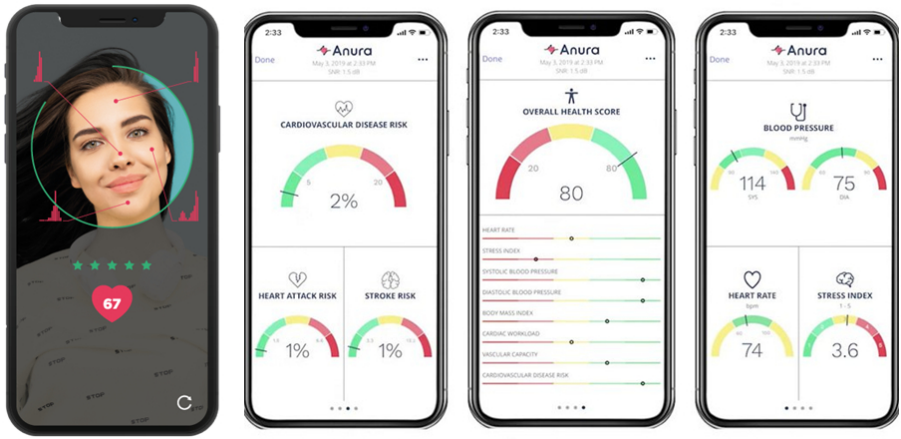
The user interface of the Anura app.

Apps using smartphones may be a solution for monitoring BP in a user-friendly way. Anura is a smartphone app based on a novel imaging methodology called transdermal optical imaging (TOI) and capitalizes on the fact that light can travel beneath the skin and be reflected due to the skin’s translucent properties [17]. The reflected light can then be captured by an optical sensor [18]. TOI uses video-captured images and machine-learning algorithms to extract blood flow information from the facial epidermis. The Anura app can make these measurements in 30 seconds by simply asking the patient to take a 30-second video selfie (Figure 1). Thus, the app may remove the need for cuffs or other technical equipment [19]. Based on a large video dataset from normotensive and hypertensive patients, along with their physiological measurements based on gold-standard medical devices, TOI uses machine-learning algorithms to build computational models to predict a variety of vital signs [20,21].

TOI technology has been shown to accurately determine BP in nonpregnant normotensive participants with a precision comparable to clinical standards using manual measurements [19]. The systolic and diastolic BP predicted from TOI resulted in measurements within 5±8 mm Hg of the reference measurements [22]. However, its performance in pregnant women remains unexplored, and the generalizability to diverse populations has not yet been established. To address this gap, the objective of this study was to evaluate the feasibility of the Anura app as a method for antenatal BP surveillance of pregnant women.

The primary aim of this study was to evaluate the reliability and accuracy of the Anura app for BP measurement during pregnancy in normotensive pregnancies, high-risk pregnancies, and in women diagnosed with preeclampsia, compared to conventional manual cuff measurements. In addition to the BP measurements, we also evaluated women’s experience of using the Anura app by a digital questionnaire.

## Methods

### Ethical Considerations

The study was conducted with ethical approval from the Swedish Ethical Review Board in Lund, Sweden (DNR 2021‐03216), and performed in line with the principles of the Declaration of Helsinki. Informed consent was obtained from all participants, who signed consent forms prior to inclusion, and no compensation was provided for participation. Participant confidentiality was strictly maintained. All data were deidentified prior to analysis, and no information that could enable identification of individual participants is presented. Data were stored securely in accordance with institutional and national data protection regulations, with access restricted to the research team.

### Design, Setting, and Participants

A total of 3 groups of pregnant women were recruited: those with normotensive pregnancies, those with high-risk pregnancies, and women diagnosed with preeclampsia. High-risk pregnancies were defined according to the Swedish Society of Obstetrics and Gynecology guidelines for hypertensive disorders in pregnancy [2]. Participants were classified as high-risk if they had at least one high-risk factor (eg, chronic hypertension, type 2 diabetes) or 3 or more moderate-risk factors (eg, advanced maternal age, nulliparity, high BMI). Preeclampsia was defined as hypertension (140/90 mmHg) after 20 weeks of gestation in combination with maternal organ dysfunction and/or intrauterine growth restriction [23,24]. The hemolysis, elevated liver enzymes, low platelets syndrome is a severe form of preeclampsia with general organ involvement, especially of the liver. Women diagnosed with preeclampsia were recruited when they were admitted to the hospital. The study was part of a prospective longitudinal feasibility study aiming to follow pregnant women with and without high-risk factors from early pregnancy until 6‐8 weeks postpartum (Multimedia Appendix 1).

The study was conducted in southern Sweden at 2 large hospital maternity health care units and 9 different antenatal health clinics (AHCs) in the region, from March 2022 to December 2023. The inclusion criteria were: (1) pregnant normotensive women, high-risk pregnancies, and women diagnosed with preeclampsia, (2) having a smartphone that could download the Anura app, (3) 18 years or older, and (4) understanding Swedish. All pregnant women enrolled in AHC during the study period were screened for eligibility and invited to participate. In total, 331 women were approached, of whom 288 agreed to participate. A total of 43 women declined participation, most commonly citing reasons such as mental illness, anxiety, concerns about having high BP, or difficulties using the Anura app. The eligible women received both oral and written information and had the opportunity to consider whether they wished to participate. They were also informed to contact their midwife or the maternal health unit if they experienced symptoms such as headache, general malaise, swelling, chest pain, or if their BP was 140/90 mm Hg or higher.

The included women were asked to use the Anura app to perform facial scans for BP measurements at each AHC or hospital visits. At the visits, the BP was also measured by midwives or health care professionals using a routinely applied manual sphygmomanometer cuff method.

The pregnancies were divided into trimesters, defined as: first trimester (0‐13+6 gestational week [GW])=0‐93 days, second trimester (14+0‐27+6 GW)=94‐187 days, and the third trimester (28+1‐42+0 GW)=188‐283 days.

### Data Collection

Demographic data for the included women (Tables1  2) was collected from the medical record system Obstetrics (version 2.18.0.100, Copyright Cerner Sverige AB) [25]. In addition to the standard antenatal program [1], the women were asked to follow a scheme for BP measurements using the Anura app. The midwives or the first author of the study provided information on downloading the Anura app to their private smartphones. Basic personal information (weight, height, age, high BP, or diabetes) was entered directly into the Anura app by the women.

**Table 1.** Demographic characteristics of included women.

Characteristic and risk factors for preeclampsia	Normotensive pregnancy, n=132	High-risk pregnancy, n=40	Preeclampsia, n=98
ASA^a^ prophylaxis, n (%)	0 (0)	35 (88)	38 (39)
High-risk factors, n (%)			
Autoimmune disease	0 (0)	1 (3)	0 (0)
IUGR^b^, IUFD^c^, placental abruption	1 (0.8)	4 (10)	6 (6)
Diabetes	0 (0)	0 (0)	6 (6)
Kidney disease	0 (0)	0 (0)	1 (1)
Chronic hypertension	0 (0)	5 (13)	13 (13)
IVF^d^ with egg donation	0 (0)	5 (13)	3 (3)
Previous GH^e^ delivery before wk 34	0 (0)	1 (3)	0
Moderate risk factors			
Maternal age (y), min-max; mean (SD)	19‐43; 31.8 (3.9)	19‐43; 35 (5.2)^f^	18‐45; 32.2 (4.8)^f^
BMI at booking visit, min-max; mean (SD)	19‐37; 24.4 (3.7)	21‐42; 29.3 (5.3)^f^	17‐61; 27.4 (6.9)^f^
African origin, n (%)	0 (0)	2 (5)	5 (5)
Nulliparity, n (%)	56 (42.7)	8 (20)	56 (57)
Family history of high BP^g^, n (%)	38 (29)	19 (48)	32 (33)
Pregnancy interval >10 y, n (%)	2 (1.5)	0 (0)	1 (1)
White coat of hypertension, n (%)	3 (2.3)	0 (0)	1 (1)
Family history of PE, n (%)	6 (4.6)	9 (23)	12 (12)
Previous PE, n (%)	0 (0)	13 (33)	18 (18)
Multiple birth, n (%)	0 (0)	0 (0)	6 (6)
Other risk factors, n (%)			
Mental illness	19 (14.5)	7 (18)	20 (20)

a ASA: acetylsalicylic acid.

b IUGR: intrauterine growth restriction.

c IUFD: intrauterine fetal death.

d IVF: in vitro fertilization.

e GH: gestational hypertension.

f *P*<.001.

g BP: blood pressure.

**Table 2.** Characteristics of included women and their newborn children

Characteristic	Normotensive pregnancy, n=132	High-risk pregnancy, n=40	Preeclampsia, n=98
Regulated BP^a^ postpartum, n (%)	1 (0.8)	11 (28)	72 (73)
Medication for hypertension at discharge, n (%)	1 (0.8)	9 (23)	65 (66)
SBP^b^, min and max; mean (SD)	100‐150; 122 (9)	120‐180; 135 (12)^c^	130-190; 156 (13)^c^
DBP^d^, min and max; mean (SD)	60‐95; 76 (8)	70‐110; 87 (9)^c^	80‐120; 98 (7)^c^
Preterm birth <wk 37, n (%)	1 (0.8)	1 (3)	36 (37)
Birth <wk 34, n (%)	0 (0)	0 (0)	21 (21)
Gestational weeks of delivery, min-max; mean (SD)	28‐42; 39.4 (1.6)	35‐41; 38.9 (1.3)^c^	26‐40; 38.81 (2.9)^c^
Vaginal birth, n (%)	101 (77.1)	26 (65)	46 (47)
Induction of labor, n (%)	36 (27,5)	18 (45)	56 (56)
Vacuum extraction, n (%)	4 (3.1)	3 (8)	4 (4)
Cesarian section, n (%)	24 (18.3)	9 (23)	51 (52)
Oxytocin augmentation, n (%)	35 (26.7)	9 (23)	16 (16)
Hemorrhage (ml), min-max; mean (SD)	100‐2100; 510 (344)	100‐3000; 568 (559)	100‐2200; 548 (429)
Twins, n (%)	0 (0)	0 (0)	9 (9)
SGA^e^, n (%)	1 (0.8)	0 (0)	27 (28)
Days in PP hospital care, min-max; mean (SD)	1‐7; 2.8 (1.3)	1‐22; 3.9 (3.6)^c^	2‐44; 9.8 (6.7)^c^
NICU^f^ admission, n (%)	11 (8.4)	2 (5)	36 (37)
Newborn weight (g), min and max; mean (SD)	1900‐4655; 3616 (477)	2550‐4438; 3480 (467)	630‐3830; 2666 (792)^c^
Newborn sex, n (%)			
Boy	60 (46)	18 (45)	49 (50)
Girl	71 (54)	18 (45)	49 (50)

a BP: blood pressure.

b SBP: systolic blood pressure.

c *P*<.001.

d DBP: diastolic blood pressure.

e SGA: small for gestational age.

f NICU: neonatal intensive care unit.

Once the app was downloaded and registered, each woman was assigned a participant code. The code key was stored under lock and key. The BPs were measured in 2 ways: by the midwife using a standard BP cuff or a validated automatic BP monitor, and by the women using the Anura app. The results were automatically stored in the Anura app. The participants manually entered the diastolic blood pressure (DBP) and systolic blood pressure (SBP) measured by the midwife into the Anura app.

In addition, the women were encouraged to measure their BP at home at least once a week, after 15 minutes of rest. For each Anura measurement, the women scanned their faces for 30 seconds at 30 frames/second imaging. The app processed the image frames and used its local algorithms to extract blood flow information. Only the extracted facial blood flow information was packaged, encrypted, and sent to the cloud server in Europe owned by NuraLogix Corporation (Toronto, Canada). The data was processed by the DeepAffex (DFX) Artificial Intelligence Engine [20]. The Anura is in full compliance with the General Data Protection Regulation. In addition, a written agreement between Lund University and NuraLogix was signed to ensure data safety, privacy, and the freedom of data use by the research team.

The women also answered a survey at 37‐39 GW. The experience of using the Anura app was investigated with a survey consisting of 8 questions. The questions concerned their perceived privacy, sense of control, security, and user-friendliness. The answer options were categorical and reported as percentages for each question.

### Data Analysis

In order to determine the number of women to be included in each group, a power calculation was performed using G*power [26]. To achieve adequate power of 80% with a significance level of 0.05 and a medium effect size (*f*=0.25), a total sample of 269 was required with 90 for each of the 3 groups (normotensive, high-risk, and preeclampsia). Data from Anura was downloaded directly from a dashboard, minimizing the risk of missing data. The clinical data were manually extracted from medical records and double-checked to ensure completeness. All analyses were performed using International Business Machines Corporation SPSS Statistics for Windows, Version 28.0. A *P* value <.05 was considered statistically significant. Demographic data were analyzed with one-way ANOVA. Bland–Altman analysis, including limits of agreement (LoA), was conducted in Microsoft Excel. All data points from the Anura app were downloaded from the NuraLogix DFX dashboard. For continuous variables, descriptive statistics were presented as minimum, maximum, and mean (SD). Categorical variables were presented in numbers (n) and proportions (%). All calculations for BP were performed separately for SBP and DBP values. The analyses were stratified by patient group and trimester. The data were normally distributed. To account for repeated measures, the 2 BP measuring methods, Anura versus manual BP, were compared using linear mixed models, fitted in R 4.3.3 using package linear and nonlinear mixed effects model version 3.1‐167. Measuring methods were defined as fixed effect and pair of measurements within person as random effect using an AR1 correlation structure. The difference in measurements is presented as the beta coefficient with 95% CI. Mixed model analyses were stratified by patient group and trimester, and assumptions were assessed graphically.

Bland-Altman plots were used to show outliers and visual comparisons of the Anura measurements against the manual cuff measurements. The Bland-Altman plot, which showed systematic differences and dispersion, did not contain any CIs but instead the 95% LoA, which were calculated assuming normal distribution. All available paired Anura BP measurements were included in the analyses. For participant questionnaires, only complete responses were included. No imputation was applied for missing data.

## Results

### Demographics

**Figure 2.**
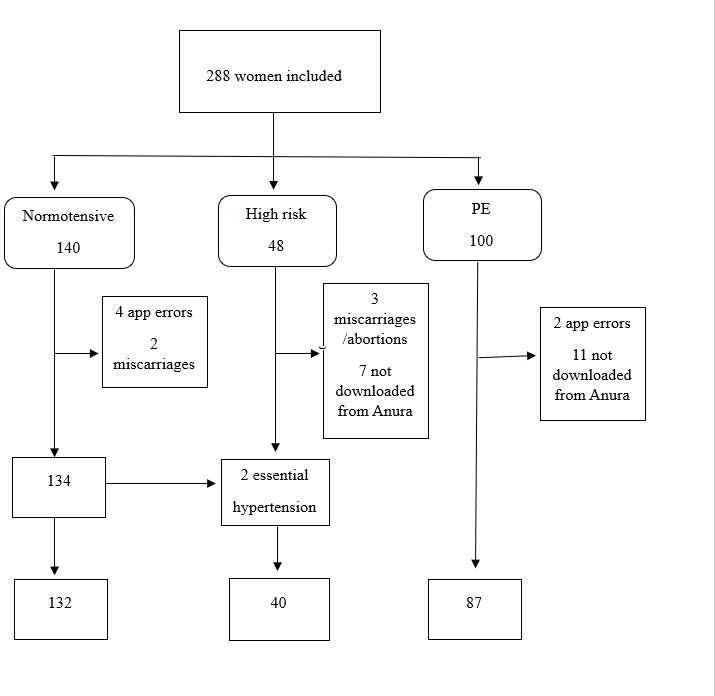
Flowchart for included women in the 3 study groups. PE: preeclampsia.

In total, 288 women were recruited for participation, but 10.1% (29/288) were excluded due to miscarriage, abortion, or technical problems with the Anura app (Figure 2). The remaining 259 women were divided into 3 groups as follows: 132 were classified as women with normotensive pregnancy, 40 were classified as those with high-risk pregnancies, and 87 were diagnosed with preeclampsia (Figure 2). Data were successfully downloaded from the NuraLogix DFX dashboard. Data could not be downloaded for 11 women with preeclampsia, but their demographic background data are still included in the preeclampsia group shown in Tables1  2, resulting in 98 for this group (Figure 2).

Demographic data showed that the normotensive pregnancy group was significantly different (*P<*.001) from both the high-risk group and the preeclampsia group regarding pregnancy length, age, height, weight, BMI, DBP, and SBP (Tables1  2). The normotensive pregnancy group had significantly lower (*P<*.001) maternal age and BMI compared to the high-risk and preeclampsia groups. Nearly half (19/40, 47.5%) of the women in the high-risk group had a family history of high BP. Additionally, most women in the high-risk pregnancy group received prophylactic acetylsalicylic acid treatment (35/40, 87.5%).

In the normotensive group, 2/140 women developed gestational hypertensions and were therefore transferred to the high-risk group (Figure 2). Of the 140 women in the normotensive group, 3 women developed preeclampsia some weeks before childbirth and were therefore transferred to the preeclampsia group. Diseases that were identified in the normotensive group were 1 case of asthma, 1 case of Crohn disease, 1 case of epilepsy, 1 case of gestational diabetes, 6 cases of hyperthyroidism, and 1 case of polycystic ovary syndrome. The fetal sex of 2 newborns remains unknown since they were born outside of the study region. Characteristics for the included women and their newborns are shown in Table 2.

In the high-risk group (n=40), 7 women developed preeclampsia, and 6 developed gestational hypertension. Eight had essential hypertension, 1 had borderline high BP, 1 had systemic lupus erythematosus or rheumatoid arthritis, and 2 had a previous history of postpartum depression. In addition, 27.5% had a regulated BP with medication postpartum, and 22.5% were still on antihypertensive medication when discharged. Women in the high-risk group also had significantly (*P*<.001) longer hospital stays.

In the preeclampsia group (n=98), 12 women had a known family history of preeclampsia and 6 had essential hypertension. Additionally, 1 woman developed hemolysis, elevated liver enzymes, and low platelets syndrome. In the preeclampsia group, 73.5% (72/98) had a regulated BP using medication postpartum and 66.3% (65/98) were still on antihypertensive medication when discharged from the maternity unit. Women in the preeclampsia group also had significantly (*P*<.001) longer hospital stays, the birthweights were significantly lower, and 36.7% (36/98) of their newborns were admitted to the neonatal intensive care unit.

### Blood Pressure Measured With the Anura App Versus Manual Measurements

In total, there were 4932 BP measurements registered in the Anura app in the 3 study groups: 2993 normotensive pregnancies, 853 high-risk pregnancies, and 1072 pregnancies with preeclampsia. Of these measurements, 539 had a corresponding manual BP cuff measurement taken at an antenatal care visit (normotensive pregnancies, n=194; high-risk pregnancies, n=108; and pregnancies with preeclampsia, n=237). Manual BP of the 3 groups and during the trimesters is shown in Table 3. For the preeclampsia group, there were few measurements in the second trimester, since most preeclampsia did not develop until the third trimester.

**Table 3.** SBP^a^ and DBP^b^ for unpaired manual measurements for each trimester.

	Manual		
Blood pressure characteristic	Normotensive pregnancy	High-risk pregnancy	Preeclampsia
Trimester 1	n=53	n=14	—^c^
SBP, mean (SD; min-max)	113 (8.9; 97‐132)	120 (11.3; 88‐135)	—
DBP, mean (SD; min-max)	69 (6.1; 54‐80)	78 (12.1; 64‐114)	—
Trimester 2	n=57	n=37	n=4
SBP, mean (SD; min-max)	115 (9.6; 97‐136)	121 (6.4; 109‐135)	156 (13.8; 140‐170)
DBP, mean (SD; min-max)	70 (5.6; 60‐83)	80 (4.8; 70‐92)	106 (9.7; 99‐120)
Trimester 3	n=74	n=50	n=185
SBP, mean (SD; min-max)	115 (9.1; 100‐134)	121 (10.2; 100‐149)	138 (10.7; 110‐180)
DBP, mean (SD; min-max)	70 (6.8; 60‐88)	77 (10.4; 60‐97)	87 (84; 63‐113)

a SBP: systolic blood pressure.

b DBP: diastolic blood pressure.

c Not applicable.

Comparisons between Anura and manual BP measurements are presented in Table 4. In the normotensive group, Anura consistently measured higher DBP across all trimesters (β ranging from 4.9 to 6.9 mm Hg; *P*<.001), while SBP tended to be lower, reaching statistical significance in the second and third trimesters. In the high-risk group, DBP was lower in the second trimester (β –3.5 mm Hg; *P*=.004), whereas SBP was significantly lower in both the second and third trimesters (β –8.4 and –9.1 mm Hg; *P*<.001). Among women with preeclampsia, Anura showed markedly lower values, with a mean difference of –6.8 mm Hg for DBP and –16.9 mm Hg for SBP (both *P*<.001).

**Table 4.** Comparison Anura—manual blood pressure measurement linear mixed effects model.

Patient type and trimester	Measurements	Individuals	DBP^a^ β (95% CI)	*P* value	SBP^b^ β (95% CI)	*P* value
Normotensive						
First	53	46	4.9 (2.8 to 7)	<.001	−2.6 (−5.6 to 0.4)	.09
Second	57	39	5.9 (4.3 to 7.5)	<.001	−3.7 (−6.6 to −0.8)	.01
Third	75	39	6.9 (4.9 to 9)	<.001	−3.2 (−5.7 to −0.7)	.01
High risk						
First	14	13	3 (−2.7 to 8.8)	.28	−2.7 (−7.3 to 1.9)	.23
Second	37	11	−3.5 (−5.9 to −1.2)	.004	−8.4 (−11.7 to −5.1)	<.001
Third	50	9	0.4 (−3.1 to 3.9)	.82	−9.1 (−12.4 to −5.9)	<.001
Preeclampsia						
Second	4	1	—^c^	—	—	—
Third	184	38	-6.8 (−8.2 to −5.4)	<.001	−16.9 (−18.7 to 15.1)	<.001

a DBP: diastolic blood pressure.

b SBP: systolic blood pressure.

c Not available.

**Figure 3.**
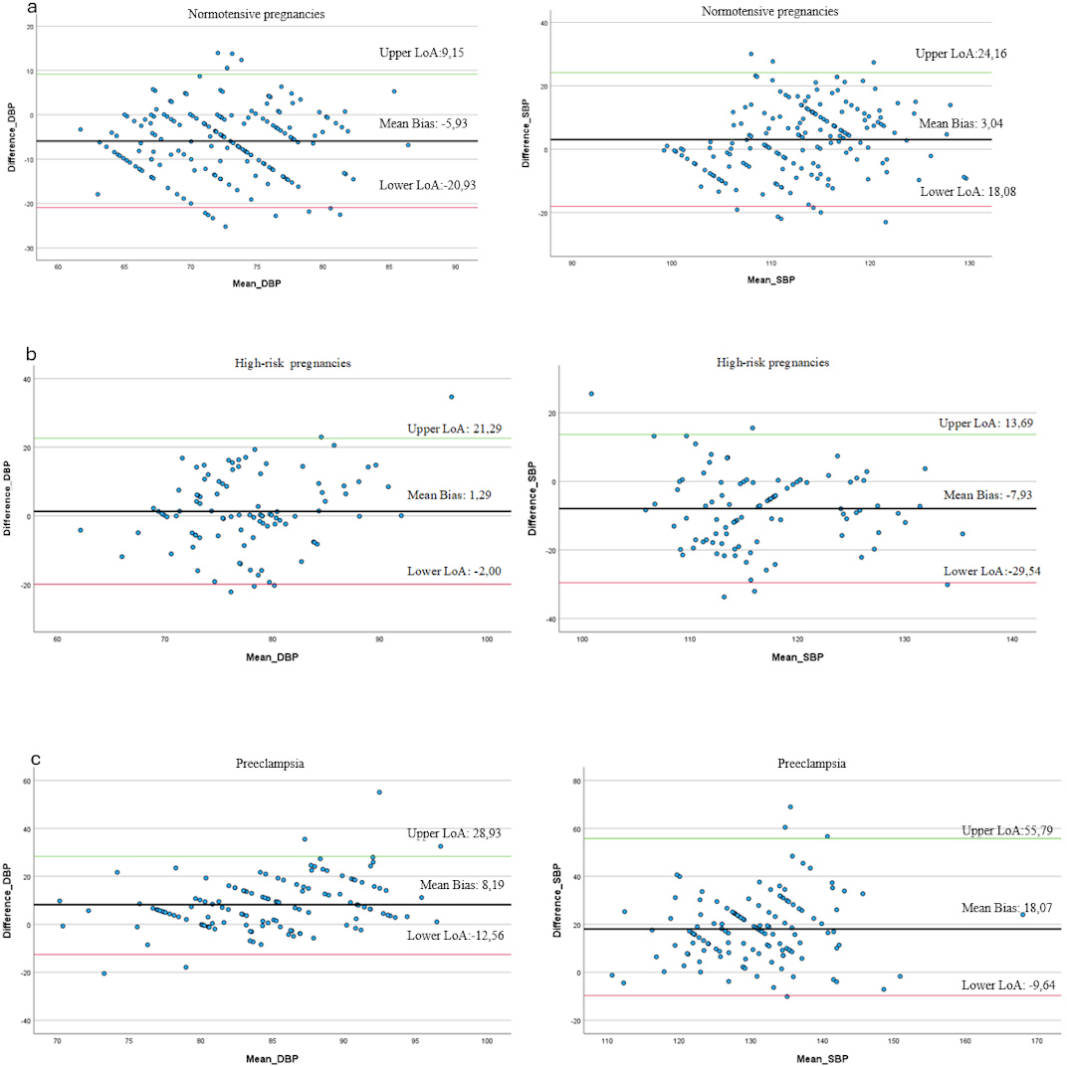
Bland-Altman plot differences in DBP and SBP for manually and Anura measurements in (a) normotensive pregnancies, (b) high-risk pregnancies, and (c) preeclampsia. DBP: diastolic blood pressure; LoA: limits of agreement; SBP: systolic blood pressure.

Bland-Altman plots (Figure 3) were created to illustrate differences between the paired BP measurements. The Bland-Altman analysis for SBP in the normotensive group (Figure 3a) shows a mean difference of 3.04 (SD 7.7) and for DBP a mean difference of −5.93 (SD 7.7). In the high-risk group (Figure 3b), the SBP shows a mean difference of −7.93 (SD 11.0) and for DBP a mean difference of 1.29 (SD 10.8). For the preeclampsia group (Figure 3c), the SBP shows a mean difference of 18.07 (SD 18.1) and for DBP a mean difference of 8.19 (SD 10.6). There was a variation in the Bland-Altman plots of the upper and lower LoA in both the SBP and the DBP in the 3 groups (Figure 3).

### The Experiences of Using Anura

The results from the survey are based on 56/172 (32.3%) women who evaluated their experience of using Anura (Multimedia Appendix 2). Most of the women (48/56, 85.7%) expressed no concern regarding their privacy while using the app, whereas 8/56 (14.3%) women reported being a little worried. Almost all women (55/56, 98.2%) reported no concern about seeing their BP results, with only 1/56 (1.8%) woman indicating slight apprehension. Regarding responsibility for their own health, 31/56 (55.4%) women felt a higher degree of responsibility when using the app. Of these, 2/56 (3.6%) women reported feeling much more responsible, 7/56 (13%) women more responsible, and 22/56 (39.3%) women a little more responsible. In contrast, 25 (44.6%) women reported no increase in responsibility. Perceived safety when using the app was also high: 51/56 (91.1%) women considered it safe, of whom 30/56 (56.6%) rated it as very safe, 21/56 (37.5%) as safe enough, and 5/56 (8.9%) women as only a little safe. Most of the women (48/56, 85.7%) experienced better control of their health with the app. Specifically, 8/56 (14.3%) women reported much better control, 18/56 (32.1%) women better control, and 22/56 (39.3%) women slightly better control. A total of 8/56 (14.2%) women reported no improvement. Similarly, 32/56 (57.1%) women reported an increased understanding of their health status, with 5/56 (8.9%) women indicating much better understanding, 17/56 (30.4%) women better understanding, and 10/56 (17.9%) women a little better understanding. A total of 2/56 (3.6%) women reported no better understanding, while 5/56 (8.9%) women remained neutral. Most women (42/56, 75%) reported that sitting still and looking into the camera during the measurement went well. However, 12/56 (21.4%) women found it unpleasant or uncomfortable, 1/56 (1.8%) woman reported it as quite unpleasant, and 11/56 (19.6%) women were neutral. The duration of the measurement was generally well accepted: 32/56 (57.1%) women considered the length just right, 22/56 (39.3%) women found it okay, and only 2/56 (3.8%) women felt it was too long.

## Discussion

### Principal Findings

The primary aim of our study was to evaluate how reliable and accurate the Anura app is for BP measurement during pregnancy, compared to manual cuffs for BP measurement. In addition, we asked women about their experiences of using the app. To the best of our knowledge, this study is the first to validate the Anura technology in pregnant women, including high-risk pregnancies and women diagnosed with preeclampsia, with analyses stratified across 3 trimesters. The use of smartphones for medical monitoring has previously indicated a need for a user-friendly, independent BP monitoring method during pregnancy to enable identification of hypertension. Home monitoring can enhance early detection [3,4] and management of pregnancy-related hypertension, while also empowering women to take an active role in their own health care [27]. This fact was also confirmed in this study, as most women reported a positive experience of using the Anura app and feelings of being in control of their own health.

The main finding in our study suggests that Anura agrees well with manual BP in normotensive women; however, discrepancies between the 2 methods increase in the high-risk and preeclampsia groups, especially for SBP. Among normotensive women, Anura generally showed a higher DBP (4.9‐6.9 mm Hg) compared to manual measurements, while SBP tended to be slightly lower, with statistical significance in the second and third trimesters. This suggests that the use of Anura BP measurement may be more reliable in women with normotensive pregnancies, while caution is required in high-risk pregnancies and once preeclampsia is diagnosed. The Bland-Altman analysis confirmed this finding, showing that the normotensive group exhibited small differences between the 2 methods, indicating that Anura has good agreement with manual measurements in normotensive women. Our findings in normotensive pregnant women are in line with previous research with male patients and nonpregnant female patients [22]. Regarding the high-risk pregnancies, the analysis showed that DBP was lower in the second trimester (–3.5 mm Hg) and SBP was lower in both the second and third trimesters (–8.4 and –9.1 mm Hg), respectively. These results show that in high-risk pregnancies, the Anura app may be less reliable in the last part of pregnancy compared to earlier stages of pregnancy. The preeclampsia group demonstrated the most pronounced differences, –6.8 mm Hg for DBP and –16.9 mm Hg for SBP. Thus, while the use of Anura BP measurement may be reliable in women with normotensive pregnancies, our results do not support using the Anura app for BP screening in women with high-risk pregnancies or women diagnosed with preeclampsia, suggesting caution in high-risk pregnancies and once preeclampsia is diagnosed. These findings are further corroborated by another study showing that BP measurements were not accurate in a perioperative setting using a similar technique, video plethysmography for contactless measurement [7]. However, Anura has been reported to be highly accurate in predicting the respiratory rate and heart rate of surgical patients in a clinical setting [7,8].

The differences shown in the Bland-Altman analysis suggest that in some cases, the Anura technology either overestimated or underestimated the BP compared to the manual measurements, especially in the preeclampsia group where the deviations were the largest. These deviations are important to consider during further processing of the variables to improve the measurements in the Anura app in the future. The high variation of the upper and lower LoA in the Bland-Altman plots may indicate that the Anura app might be less reliable at extreme BP values, or that its performance varies depending on the context or conditions of the measurements [28]. One possible explanation is that women with high-risk pregnancies and preeclampsia may have more edema in their faces, which could interfere with the TOI technique that fails to make accurate BP measurements. A physiological explanation for this inconsistency in BP may lie in the cardiovascular adaptations that occur during pregnancy. Normally, the blood volume increases during the first 2 trimesters, and the peripheral vascular resistance decreases [29], leading to a 10‐20 mm Hg reduction in BP. It reaches its lowest point at 18‐20 weeks of gestation and then returns to prepregnancy levels by the third trimester [30,31]. In preeclampsia, however, there is a maintained vascular resistance, as well as endothelial damage, that results in general edema. Increased edema and hemo-concentrated blood might impact the measurement quality when BP is measured using TOI. Our results indicate that the Anura measurements tended to show lower values compared to manual BP measurements for both DBP and SBP in the preeclampsia group, and to some extent also in the high-risk group. In preeclampsia, the vascular endothelial cells undergo changes that result in inflammation, impaired blood flow regulation, and increased vascular permeability [10,32]. While most pregnant women experience swelling during their pregnancy, this predominantly does not occur until the third trimester in normotensive pregnancies [33].

In previous studies, BP was determined in nonpregnant individuals by Anura with high accuracy [19,22]. In this study, the technology was evaluated under controlled conditions with optimal lighting, a tripod-mounted mobile phone, and using the same device for all measurements. In contrast, this study allowed the women to use their own mobile phones, holding them manually, and having varying lighting conditions at home. Movement and lighting often affect the accuracy of contactless measurement technologies using a mobile phone [21,34]; yet, while knowing their role for accurate BP measurement, we could neither entirely control nor account for them in this study. The women were instructed to rest in a sitting position and hold their mobile phones still at arm’s length in front of their faces during the 30 seconds needed for measurements. They were to measure their BP only when at least 3 stars appeared in the Anura app. One star indicates poor lighting conditions, while 5 stars indicate perfect lighting conditions [35]. Any movements of the arm and varying light qualities in the rooms at AHC and at home could, of course, affect the measurements. This may apply particularly to women admitted to the hospital with preeclampsia, who often had to dim the lights in their rooms due to symptoms and illness related to preeclampsia. Such suboptimal conditions may have impacted the performance of the Anura measurements. However, our results contribute to the advancement of contactless BP monitoring by highlighting the challenges of implementing this technology. These insights can be used to further improve the handling of the smartphone, lighting conditions, and the algorithm in the future.

The use of smartphones for medical monitoring has increased in recent years. As information technology continues to develop, so do the opportunities to find new ways to measure BP with smartphone-based apps [36]. Compared to traditional monitoring, home BP monitoring during hypertensive pregnancies appears to be cost-saving without compromising the safety of the pregnant mother [37]. It increases accessibility and convenience for pregnant women, has the potential to decrease the number of hospital visits [3], enables the early detection of abnormalities, and promotes better control and self-management of their health. It has also been shown that BP measured at home often is lower than in a clinical setting [38]. In addition, home measurements have shown reduced stress and anxiety and enable more individualized care, which in turn can lead to better health outcomes for the women and their children. In a systematic review and meta-analysis [39], it was demonstrated that contactless monitoring technology using consumer-friendly cameras, such as smartphones, is accurate for measuring heart rate compared to other medical devices. However, more studies are needed to assess the accuracy of contactless BP measurements, particularly among pregnant women. In another review, it was also highlighted that no apps for BP had undergone sufficient testing to be recommended in a clinical practice [40]. In a recently published study, the accuracy of contactless monitoring technology for heart rate suggested limitations in accurately measuring BP in a hospital setting. However, the study showed that contactless monitoring technology was both accurate and feasible for measuring respiratory rate in a hospital setting [7]. In an earlier study using Anura, the SBP and DBP predicted from TOI fell within 5±8 mm Hg of reference measurements [22], but in our study, these criteria were not met in the paired data for the 3 groups. In yet another study using Anura, it was also shown that TOI can determine heart rate, heart rate variability, and infer stress of an individual with high accuracy [21]. Given the Anura precise measurement of heart rate and stress, the app remains an interesting tool for assessing BP. By leveraging advanced machine learning techniques and enhancing the app’s sensitivity, those responsible for the app may expand its use and reliability.

In this study, most women expressed a positive experience of using the Anura app. They also reported a higher degree of responsibility and control over their health. This agrees with another study that also found the participants to be satisfied with the contactless technology and would recommend it for future clinical settings [7]. The women generally perceived the app as adequate and safe for use, with nearly all women reporting no elevated anxiety when seeing their BP results. Concerns were raised by some women regarding the accuracy of the measurements and the handling of their data, particularly in relation to privacy. These findings suggest that the app has the potential to be well-received by women during pregnancy, though addressing these concerns will be important for its future use.

### Limitations and Future Solutions

A key strength of the study is the use of linear mixed models to account for repeated BP measurements within individuals, which provides a robust estimation of differences between the Anura app and manual measurements compared to simpler paired analyses. Stratifying analyses by patient group and trimester allowed us to explore potential variations across different risk profiles and gestational stages. An additional strength is that the analyses have been quality assured through independent review by a statistician.

However, some limitations should be acknowledged. The models did not adjust for potential confounding factors such as maternal age, BMI, or comorbidities, which may influence BP variability and limit the generalizability of the findings. We also acknowledge a lack of insight into the validation of the underlying technology. Our study sought to explore the use of Anura in a clinical setting with pregnant women, with the primary aim to compare measurement methods. With this aim, and the paired design, we see no benefit to adjust the analysis for maternal age, gestational age, or comorbidities. Additionally, the study focused on comparing measurement methods rather than evaluating clinical outcomes, so the diagnostic accuracy and predictive value of the Anura app for conditions such as preeclampsia remain unassessed. The sample sizes in some subgroups, particularly patients with high-risk pregnancies and preeclampsia in early trimesters, were relatively small, which may reduce statistical power and precision, particularly regarding high-risk pregnancies. Due to these limitations, future studies with larger sample sizes and better compliance are needed to further validate these observations in a clinical setting. Finally, while mixed models handle within-subject correlation, physiological variability and measurement conditions may still contribute to residual variability that is not fully captured by the models. Furthermore, while the Anura app has previously shown a measurement error of approximately 5±8 mm Hg [22], we did not conduct a formal error assessment or apply corrections in the present study. We acknowledge this as a limitation and emphasize that our findings should be interpreted as preliminary feasibility results rather than accuracy validation.

Another limitation was that only 32.6% of the women answered the questionnaire evaluating their experiences of using the Anura app, so caution is advised when interpreting these results. Completing surveys through an app shortly before giving birth may not have been an ideal time point for the women. Additionally, we were unable to send reminders within the app. To improve compliance, future studies might consider alternative methods, such as providing the questionnaires directly by the midwife at the AHC or calling the women by phone to gather their perspective on the experience.

Our results are partly in line with previous studies that reported both acceptable accuracy and significant margins of error for noncontact BP measurements [6]. Discrepancies can be partly explained by differences in study design, population, and measurement conditions, and previous reviews point to methodological limitations, especially in studies of pregnant women [5]. We also acknowledge that Anura was not compared to 24-hour ambulatory BP monitoring in this study, and we note this as a limitation. Future studies should include such comparisons to better evaluate performance across all risk groups. Home BP measurements offer numbers of benefits [3,4,27] and the ability to measure cuffless BP in a home setting appears to be a promising solution for pregnant women in the future.

Overall, the analyses provide useful insights into the feasibility and agreement of the Anura app with standard BP measurements, but future studies incorporating larger samples, clinical outcomes, and adjustments for relevant confounders are needed to more definitively assess its clinical use. Future research should evaluate Anura in larger and more diverse populations, including women with chronic hypertension and multiple comorbidities. Longitudinal studies are needed to assess performance across pregnancy. Beyond BP monitoring, the technology may enable early detection of other pregnancy-related complications. Usability, integration with clinical systems, and data security should also be explored to support safe implementation in AHC.

### Conclusion

The Anura app showed a good acceptance among women with normotensive and high-risk pregnancies. They experienced increased security and control over their health. In normotensive pregnancies, the Anura BP showed a good accuracy; however, the reliability was limited in high-risk and preeclampsia groups, especially for the SBP. The app can therefore currently only be considered as a supportive complement in normal pregnancy, but not a substitute for validated clinical equipment. Continued development and adaptation of algorithms, with data from high-risk pregnancies, is necessary to enable broader clinical use. Such development could strengthen preventive work and contribute to better care for women at risk of hypertensive pregnancy complications.

## Supplementary material

10.2196/70370 Multimedia Appendix 1 Study protocol for the Anura study.

10.2196/70370 Multimedia Appendix 2 Responses to the questionnaire on women’s experiences of using the Anura app.

## Abbreviations

AHC: antenatal health clinic
BP: blood pressure
DBP: diastolic blood pressure
DFX: DeepAffex
GW: gestational week
LoA: limits of agreement
NICU: neonatal intensive care unit
SBP: systolic blood pressure
TOI: transdermal optical imaging
