# Supplementary material for: Evaluating a Smartphone App to Monitor Blood Pressure in Normotensive Pregnancies, High-Risk Pregnancies, and Women With Preeclampsia: Prospective Longitudinal Feasibility Study
Source: JMIR Hum Factors. 2026 Feb 18;13:e70370. doi: 10.2196/70370 (PMC12916089; doi:10.2196/70370)
Supplement: Multimedia Appendix 1 [file humanfactors-v13-e70370-s001.docx]

**Study protocol for the Anura study**

We estimate to include (n = 100) women with a high-risk pregnancy (defined as those eligible for prophylactic treatment with ASA), (n = 100) healthy pregnant women as controls and (n=100) women with preeclampsia (PE).

The inclusion criteria are:

- Pregnant healthy women at the Antenatal Health Care (AHC) units.
- Pregnant women with high-risk pregnancies at the AHC units.
- Women with PE at the hospital units.
- Women who have a mobile phone that can download the application ANURA.
- Women who are 18 years or older.
- Women who understand Swedish.

All women invited to participate in the study will be asked these questions to ensure correct group allocation. Women who meet at least one criterion in the first table, or three or more criteria in the second table, will be included in the high-risk group. At least one of the following criteria is fulfilled:

| Criterion | YES | NO |
| --- | --- | --- |
| Previous preeclampsia, regardless of severity |  |  |
| Previous gestational hypertension with delivery before gestational week 34+0, or fetal growth restriction, or IUFD, or placental abruption |  |  |
| Chronic hypertension |  |  |
| Diabetes (type 1 or 2) prior to pregnancy (not gestational diabetes) |  |  |
| Autoimmune disease such as SLE or APS (antiphospholipid syndrome) |  |  |
| Chronic kidney disease, proteinuria at booking |  |  |
| Multiple pregnancy |  |  |
| IVF with egg donation |  |  |

Three or more of the following criteria are fulfilled:

| Criterion | YES | NO |
| --- | --- | --- |
| Primiparity |  |  |
| BMI ≥ 30 |  |  |
| Age ≥ 40 years |  |  |
| Family history of preeclampsia (mother or sister) |  |  |
| Interpregnancy interval > 10 years |  |  |
| sBP > 130 mmHg or dBP > 80 mmHg at booking |  |  |
| African origin |  |  |
| Physician-diagnosed obstructive sleep apnea |  |  |
| “White coat hypertension” (repeatedly elevated BP when measured in health care but normal BP recorded at home) |  |  |

Time-points for blood pressure measurement with ANURA:

During the first visits to the midwife at AHC (week 8-14)

At each consecutive visit at AHC

At least once a week at home in the pregnancy

When the blood pressure is taken by the medical staff at the hospital

Time-points for surveys:

Pregnancy week 37-39. (evaluation experiences of the app ANURA)

| **Pregnancy**  **week** |  | 8 | 9 | 10 | 11 | 12 | 13 | 14 | 15 | 16 | 17 | 18 | 19 | 20 | 21 | 22 | 23 | 24 | 25 | 26 | 27 | 28 | 29 | 30 | 31 | 32 | 33 | 34 | 35 | 36 | 37 | 38 | 39 | 40 |
| --- | --- | --- | --- | --- | --- | --- | --- | --- | --- | --- | --- | --- | --- | --- | --- | --- | --- | --- | --- | --- | --- | --- | --- | --- | --- | --- | --- | --- | --- | --- | --- | --- | --- | --- |
|  |  |  |  |  |  |  |  |  |  |  |  |  |  |  |  |  |  |  |  |  |  |  |  |  |  |  |  |  |  |  |  |  |  |  |
| Normotensive | n=100 | The women are enrolled in maternity care | 1 |  |  |  |  |  |  |  |  |  |  |  |  |  |  |  |  |  |  |  |  |  |  |  |  |  |  |  | 2 |  |  |  |
| High-risk | n=100 |  |  |  |  |  |  |  |  |  |  |  |  |  |  |  |  |  |  |  |  |  |  |  |  |  |  |  |  |  |  |  |  |  |
|  |  | Blood pressure is taken at every visit. | 1 |  |  |  |  |  |  |  |  |  |  |  |  |  |  |  |  |  |  |  |  |  |  |  |  |  |  |  |  |  |  |  |
| Preeclampsia | N=100  3-4% from the patients above. The rest from the hospital at diagnosis |  |  |  |  |  |  |  |  |  |  |  |  |  |  |  |  |  |  |  |  |  |  |  |  |  |  |  |  |  |  |  |  |  |

1. Blood pressure with Anura and manual cuff blood pressure

2. Responding to a survey about experiences using Anura
