# Supplementary material for: Evaluating a Smartphone App to Monitor Blood Pressure in Normotensive Pregnancies, High-Risk Pregnancies, and Women With Preeclampsia: Prospective Longitudinal Feasibility Study
Source: JMIR Hum Factors. 2026 Feb 18;13:e70370. doi: 10.2196/70370 (PMC12916089; doi:10.2196/70370)
Supplement: Multimedia Appendix 2 [file humanfactors-v13-e70370-s002.docx]

# **Multimedia appendix 1**. Responses to the questionnaire on women’s experiences of using the Anura™ application (N = 56).

| **Survey question**s | Response options | **N** (%) |
| --- | --- | --- |
| 1. Were you worried about your privacy while using the Anura application? | Not concerned at all  A little worried | 48 (86)  8 (14) |
| 2. How did you experience seeing your blood pressure in the application? | Wasn't worried at all  Was slightly worried | 55 (98)  1 (2) |
| 3. Did you feel more responsible for your health when using the application? | Much more responsibility  More responsibility  A little more responsibility  No more responsibility | 2 (4)  7 (13)  22 (39)  25 (45) |
| 4. Did it feel safe to use the application and measure your blood pressure? | A little safe  Safe enough  Very safe | 5 (9)  21 (38)  30 (54) |
| 5. Did you experience better control of your health with the application? | Much better control  Better control  Slightly better control  No better control | 8 (14)  18 (32)  22 (39)  8 (14) |
| 6. Did you experience increased understanding of your own health with the application? | Much better understanding  Better understanding  Little better understanding  No better understanding  Neutral | 5 (9)  17 (30)  10 (18)  2 (4)  5 (9) |
| 7. What did you think of sitting still and looking into the camera during the measurement? | Going well  It is quite unpleasant/uncomfortable  It is unpleasant/uncomfortable  Neutral | 42 (75)  1 (2)  12 (21)  11 (20) |
| 8. What do you think about the length of time it takes to measure blood pressure in the application? | It's just the right length  It's pretty okay  It takes too long | 32 (57)  22 (39)  2 (4) |
